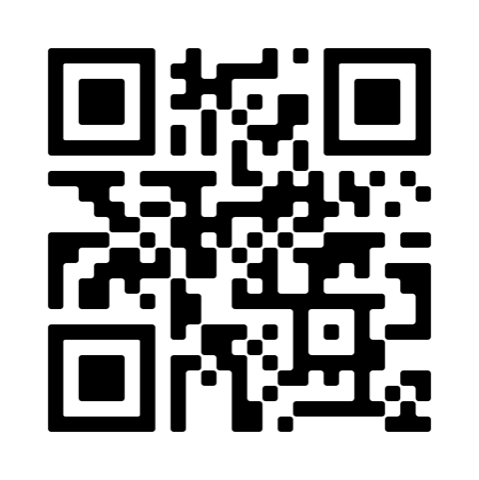# The first European gynaecological procedure with the new surgical robot Hugo™ RAS. A total hysterectomy and salpingo-oophorectomy in a woman affected by BRCA-1 mutation

**DOI:** 10.52054/FVVO.14.1.014

**Published:** 2022-04-03

**Authors:** G Monterossi, L Pedone Anchora, S Gueli Alletti, A Fagotti, F Fanfani, G Scambia

**Affiliations:** UOC Ginecologia Oncologica, Dipartimento per la salute della Donna e del Bambino e della Salute Pubblica, Fondazione Policlinico Universitario A. Gemelli IRCCS, Rome, Italy; UOC Ginecologica e Ostetricia, Dipartimento Materno-Infantile, Ospedale Buccheri La Ferla Fatebenefratelli, Palermo, Italy; Università Cattolica del Sacro Cuore, Rome, Italy

**Keywords:** HUGO RAS, total hysterectomy, robotic surgery

## Abstract

**Background:**

The benefits of minimally invasive surgery are well known in gynaecology. Robotic-assisted surgery has gained widespread acceptance within the surgical community and seems to be the most rapidly developing sector of minimally invasive surgery.

**Objectives:**

This video shows the salient steps of total hysterectomy with new robotic technology, Hugo™ RAS. The objectives were to introduce and demonstrate the feasibility, efficacy, and safety of this new advanced device.

**Materials and Methods:**

A sixty-two years-old woman affected by BRCA-1 mutation underwent the first European gynaecological surgical procedure using the new surgical robot Hugo™ RAS in the Division of Gynecologic Oncology, Fondazione Policlinico Universitario A. Gemelli IRCCS, Rome, Italy.

**Main outcome measures:**

Docking and operative times.

**Results:**

The docking time was 6 minutes and the total operative time was 58 minutes. There were no system errors and faults in the robotic arms. The surgeon found no friction or rasping in the arms. The estimated blood loss was 30 mL. No intraoperative complications were recorded.

**Conclusion:**

Gynaecological surgery with Hugo™ RAS seems feasible, safe and effective as shown by initial experiences in urological surgery. A larger case series would confirm the current experience and determine whether this technology could offer any additional benefit.

## Learning objective

Hugo™ RAS is a new robotic technology for minimally invasive abdominal surgical treatment. It has been used in the gynaecological field to perform a total hysterectomy with bilateral salpingo- oophorectomy. This video shows the salient steps of the procedure that underline the feasibility, efficacy, and safety of this new surgical tool.

## Introduction

The benefits of minimally invasive approach are well known in gynaecology (Kluivers et al., 2007; Aarts et al., 2015). The technological innovations of the robotic surgery allowed extending minimally invasive surgery to even the most complex cases so that in recent years there has been a further increase in the rate of minimally invasive surgery (Gressel et al., 2020).

Robotic-assisted surgery has gained widespread acceptance within the surgical community and seems to be the most rapidly developing sector of minimally invasive surgery.

Although the Da Vinci® (Intuitive Surgical) represented the leading actor in defining the “rules” of robotic surgery, technology continues to move forward and new competitors have been developed over the last few years (Haig et al., 2020; Fanfani et al., 2015; Fanfani et al., 2016a). Amongst these, the most recently introduced robotic system is the Hugo™ RAS Technology manufactured by Medtronic. It is composed of a system tower, an open console and four arm carts. Each robotic arm is independent, allowing the placement of the robotic arms from all directions in order to reduce risk of collision; moreover it has a high range of movements enabled by six different joints per arm. The surgeon performs procedures from an “open” surgical console composed of a 32-inch-wide screen HD-3D passive display, two arm-controllers with handgrip similar to the pistol grip and a footswitches panel to control the camera, energy sources, and the reserve arm.

## Patients and methods

A sixty-two year-old woman affected by BRCA-1 mutation underwent the first European gynaecological surgical procedure by the new surgical robot Hugo™ RAS at the Division of Gynecologic Oncology, Fondazione Policlinico Universitario A. Gemelli IRCCS, Rome, Italy.

She was Caucasian, with a body mass index of 28. The patient gave a history of two vaginal deliveries without complications and an appendectomy during childhood. Preoperative evaluation by pelvic ultrasound showed a normal uterus and adnexae. The CA 125 level was <30 µg/mL.

After giving an informed consent, patient underwent prophylactic total extra-fascial hysterectomy with bilateral salpingo-oophorectomy. The total hysterectomy was performed step by step with uterine arteries ligation at the origin according as described previously (Gueli Alletti et al., 2019).

At the end of the procedure an intra-peritoneal drain was placed.

During the surgical procedure, specific time parameters were assessed:

- docking time, defined as the time between the placement of all trocars and the actual start of the robotic part of the procedure at the complete positioning of all robotic arms and instruments.
- operative time, defined as the interval from the start of the procedure to the suturing of the surgical incisions.

Postoperative pain evaluation during the immediate postoperative period was recorded at 2, 4, 12, and 24 h after surgery, using a validated visual analogue pain scale (VAS) and scored from 0 to 10 (0 = no pain;10 = agonising pain) (McCormack et al., 1988).

The duration of the hospital stay was calculated from the day of surgery (day 0) to discharge.

## Results

Under general anaesthesia, the patient was positioned in the dorsal lithotomy position with both legs supported in Allen stirrups with a Trendelenburg tilt and arms along the body.

The patient received antibiotic prophylaxis consisting of cefazoline 2g administered intravenously 1 hour before surgery and antithrombotic prophylaxis consisting of enoxaparin 4000 IU subcutaneously once a day from the day after the surgery.

The adjustable robotic arms were a maximum of four and could be individually positioned in different arrangements in space, detached from each other. In our setting, we decided to use three robotic arms, one for the endoscope and the other two for three different instruments: bipolar fenestrated grasper on the left arm, monopolar curved scissors on the right arm and, during the vault closure, a large needle driver on the right arm after removal of curved scissors.

**Figure 1:**
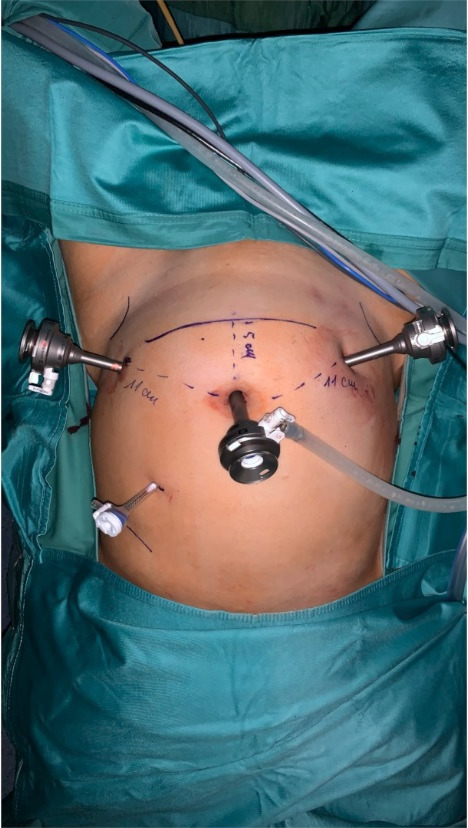
External view of trocars placement.

**Figure 2:**
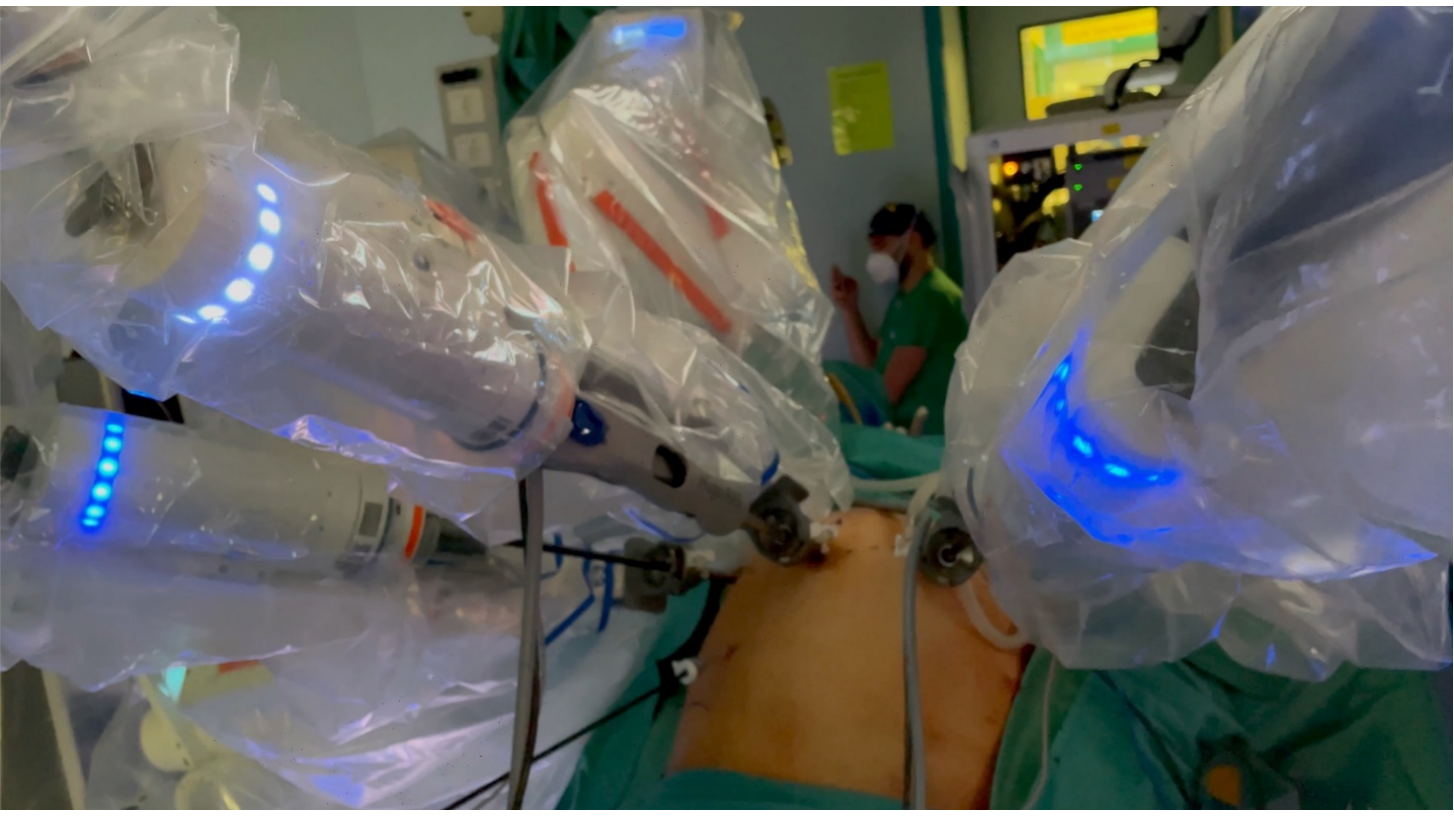
The robotic arms after docking.

We used four ports to perform the surgical procedure; an umbilical port for the 11 mm optics (arm number 1) was first inserted, the second (arm number 2) and third (arm number 3) access points were made using 8 mm titanium trocars in the left and right iliac fossae, 11 cm of distance from the umbilical port. Fourth access point was a 5mm trocar in the Palmer’s point, which was used by the table assistant for suction and irrigation, grasping, and sealing the uterine arteries at their origin with a vascular clip. (Figures 1 and 2) The first surgeon from the console controlled the movement of both instruments and the camera. The first assistant was situated at the patient’s left side. The second assistant placed and moved the uterine manipulator.

The docking time was 6 minutes and the total operative time was 58 minutes. There were no system errors or faults in the robotic arms. The surgeon found no friction or rasping in the arms.

The estimated blood loss was 30 mL. No intraoperative complications were recorded.

Pelvic drain was removed the day after surgery. Pain VAS score decreased after surgery, with 2 - 4 -12 hours values of 4 – 4 -2, respectively. At 24 h hours the value of pain was 2. The patient was discharged on the second postoperative day.

## Discussion

Robotic surgery is an area in which technological development is contributing significantly to the improved patient care. In this video article we present the first clinical experience with this new technology in gynaecological surgery and demonstrate encouraging outcome. One of the main concerns when using a new advanced device is the occurrence of errors or system crashes that could force restarting the system or even abandoning the robotic approach with laparoscopic or laparotomic conversion. This could have a negative impact on the patient with increased risk of complications and prolonged the operative time.

During the present operation, the Hugo™ RAS system showed fluidity and promptness of response to the commands of the first surgeon, both in terms of arm and instrument movements and activation of the energy sources so that good results in operative time and blood loss were achieved.

Having independent arm carts, as it is in other robotic systems (Haig et al., 2020; Fanfani et al., 2016b; Rossitto et al., 2016; Rossitto et al., 2017), has both advantages and disadvantages. Trocar placement could be modified according to the scheduled procedure without limitations, since it is not necessary that all the arms must be coming from the same direction. For the present case we decided to place lateral trocars similar to standard laparoscopy, a little lower than the umbilical trocar. This ensured a better aesthetic result and allowed a more ergonomic placement of the trocars in case of a possible laparoscopic conversion.

Moreover, independent arms allowed more movement and fewer clashes. As a matter of fact, no intra- or extra-abdominal collisions occurred during the procedure. On the other hand, having four independent arms means a larger footprint around the patient and storage space would be required compared to other systems.

However in the present surgery the first assistant and the nurse maintained a comfortable position during the procedure without limitation of their movements. With the wrist-like articulation of the instruments, along with the rotation multiplier technology, it was easy to reach the less accessible anatomical points and to have an optimal angle for effective coagulation. Moreover, suturing was facilitated by amplifying the rotation of the surgeon’s wrist. The first surgeon maintained an ergonomic position during the procedure due to the open console. Moreover, avoiding the need to look into a “closed” display allowed an easy communication between the first surgeon and the team along with a direct view of the surgical field. In the present case the operative time was shorter than those reported for hysterectomy performed by Da Vinci ® system in clinical trials and superimposable to those reported for standard laparoscopy (Albright et al., 2016). Blood loss was also similar to the mean reported in cases of hysterectomies performed by Da Vinci® system or standard laparoscopy.

## Conclusions

Although it is too early to reach definitive conclusions, gynaecological surgery with Hugo™ RAS seems feasible, safe and effective in a similar way to the initial experience in urological surgery (Ragavan et al., 2022). A larger case series would confirm the experience and allow determining whether this technology could offer additional benefit. A clinical prospective study is already underway in our centre in order to provide further evidence.

## Video scan (read QR)

https://vimeo.com/688801633/a39465e360

**Figure:**